# The serum response factor/cofactors interactions and their implications in diseases

**DOI:** 10.1111/febs.15544

**Published:** 2020-09-03

**Authors:** John Oloche Onuh, Hongyu Qiu

**Affiliations:** Center for Molecular and Translational Medicine, Institute of Biomedical Science, Georgia State University, Atlanta, GA, USA

**Keywords:** binding, cofactors, mechanisms, myocardin, serum response factor, transcription

## Abstract

Serum response factor (SRF), a member of the *Mcm1, Agamous, Deficiens, and SRF* (MADS) box transcription factor, is widely expressed in all cell types and plays a crucial role in the physiological function and development of diseases. SRF regulates its downstream genes by binding to their CArG DNA box by interacting with various cofactors. However, the underlying mechanisms are not fully understood, therefore attracting increasing research attention due to the importance of this topic. This review’s objective is to discuss the new progress in the studies of the molecular mechanisms involved in the activation of SRF and its impacts in physiological and pathological conditions. Notably, we summarized the recent studies on the interaction of SRF with its two main types of cofactors belonging to the myocardin families of transcription factors and the members of the ternary complex factors. The knowledge of these mechanisms will create new opportunities for understanding the dynamics of many traits and disease pathogenesis especially, cardiovascular diseases and cancer that could serve as targets for pharmacological control and treatment of these diseases.

## Introduction

Serum response factor (SRF) is a member of the *Mcm1, Agamous, Deficiens*, and *SRF* (MADS) box transcription factor widely expressed in all cell types. SRF participates in multiple biological functions in many cells, such as muscle cells (cardiac, skeletal, and smooth), endothelial cells, fibroblasts, hepatocytes, immune cells, and neurons [1-3], and plays a crucial role in the tissue development of gastrointestinal tracts (GI), and cardiovascular and immune systems [4-8]. SRF is also involved in various diseases’ pathogenesis, including multiple types of cardiovascular diseases (CVD) and cancers [1,9].

Although the importance of SRF in these conditions is widely recognized, the mechanisms involved remain largely unknown and need to be investigated. Several studies have suggested that the CArG box [CC(A/T)_6_GG] DNA sequences within the promoters of some genes are critically responsible for the transcriptional effects of SRF [1,2,10]. CArG element is a component of the serum response element (SRE) that is present in the promoter of *c-fos*, one of the immediate early genes (IEG) [11]. CArG box is vital for the process of serum induction of the promoter when stimulated by growth factors. It is commonly known as the consensus binding site for the SRF [11]. For example, evidence indicates that the binding between SRF and CArG box is vital for the expression of smooth muscle cell (SMC) genes that mediate the cellular differentiation and proliferation under physiological conditions and also play critical roles in the development of vascular diseases [11]. Importantly, studies have demonstrated that the transcription effect of SRF on these downstream genes relies on its interaction with the diverse cofactors to constitute a functional SRF/cofactor complex controlling the downstream gene expression [1,11].

This SRF/cofactor interaction varies depending upon different stimuli and mediates a distinct effect in a cell-specific manner, resulting in a high diversity of SRF functions. Due to the importance of these regulations of SRF in both physiological and pathological conditions, increasing attention has been focused on this research area. Numerous studies are being conducted to reveal the underlying mechanisms responsible for the SRF/cofactors interaction and their potential roles in the pathogenesis of the diseases [1].

In this review, we summarized the new progress in the studies related to the effects of SRF, focusing on the molecular basis and regulatory mechanisms of the interaction between SRF and its main cofactors. With a comprehensive search of the PubMed database, we collected the published articles on SRF/cofactor interactions and the health outcomes, especially in CVD and cancers. Specifically, we mainly included the recent studies and highlighted the new information in the following aspects; first, we discussed the molecular basis of SRF and its main cofactors, as well as the regulations of their interaction; Secondly, we highlighted the effects of SRF/cofactors at multiple levels, including the molecular level on the expressions of the downstream genes, cellular functions, tissue development, and physiological function. Thirdly, we presented the implications of SRF/cofactors interactions on various diseases focusing on CVD and cancers. Also, we presented the perspectives on future research direction on the related areas.

## The main cofactors of SRF

**Fig. 1.**
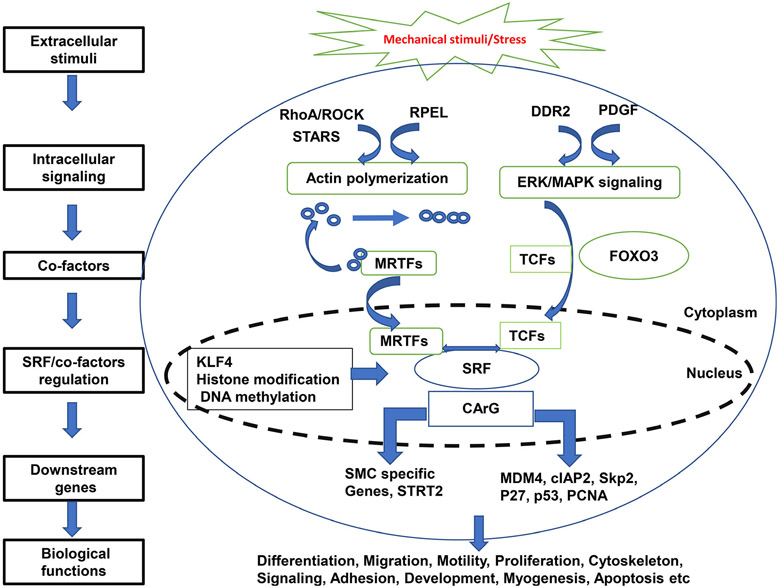
Overview of the molecular mechanisms governing SRF binding to cofactors and the subsequent transcription of target genes in cells. SRF, serum response factor; ROCK, RhoA kinase; STARs, striated muscle activator of Rho signaling; MRTFs, myocardin families of transcription factors; DDR2, discoidin domain receptor 2; PDGF, platelet-derived growth factor; ERK, extracellular signal-regulated protein kinase; MAPK, mitogen-activated protein kinase; TCFs, ternary complex factors; FOXO3, forkhead box O3; KLF4, Kruppel-like transcription factor 4; MDM 4, Mouse Double Minute 4 protein; cIAP2, cellular inhibitor of apoptosis protein 2; Skp2, S-phase kinase-associated protein 2; PCNA, proliferating cell nuclear antigen.

Serum response factor regulates numerous gene expressions through its association with various accessory cofactors, among which the most well-reported ones are myocardin-related transcription factors (MRTFs) and the members of the ternary complex factors (TCFs) [2,3,11] (Fig. 1). Although other potential cofactors, such as GATA and NK2 homeobox 5 family of transcription factors, are reported, they are involved to a lesser extent [12].

Myocardin-related transcription factors, including myocardin, MRTF-A/MKL1/MAL, and MRTF-B/MKL2, comprise a family of related transcriptional co-activators with multiple biological functions which appear in other reviews [2,13]. MRTFs physically associate with SRF and synergistically activate transcription, which regulates cellular differentiation by activating the downstream genes through their interactions with the CArG box [11,14-16]. The expression of myocardin is specific to the cardiac and vascular SMCs, while MKL1 and MKL2 are more broadly expressed [3,11]. By interacting with SRF, myocardin induces the expression of SMC marker genes. At the same time, the MKL1 and MKL2, on the other hand, are involved in actin dynamics resulting in the control of SMC-specific contractile genes during actin polymerization [3,17]. However, they do not bind directly to DNA sequences but associate directly with SRF to control SMC gene transcription [3,18]. SRF connects with the Rho-actin cytoskeleton to initiate transcription response through its binding interactions with the MRTFs [19,20]. This can also alternatively be made possible in response to cell proliferative growth factor stimulation with the consequent displacement of myocardin in favor of ETS like-1 protein (Elk-1) interaction and the increased expression of IEGs, for instance, *c-fos* [21,22].

The TCFs, including the ETS-like proteins (Elk1, Elk3, and Elk4), associate with SRF through the mitogen-activated protein kinase (MAPK or MAP kinase) in response to serum or growth factors and regulate IEGs [3,13,19,23]. Interactions between the TCFs and SRF result in a simultaneous binding to ETS-binding site adjacent to the CArG box [3]. Also, the TCFs have been reported to have the capacity to be independent of SRF [24-26].

The MKLs and TCFs interact with SRF in a mutually exclusive manner and compete for SRF DNA-binding domain. MKLs are recently known to be involved in the regulation of some IEG expressions under serum induction [3,13,17,27]. Some IEGs have been reported to be coupled to one pathway or another in fibroblasts, while in SMCs, platelet-derived growth factor (PDGF) induces cofactor exchange [22,24]. However, it is uncertain whether the cofactor competition is commonly associated with SRF regulation *in vivo*.

Numerous studies have implicated the TCFs in cell proliferation and cancer; however, the extent to which the transcription of IEGs is TCF-dependent and the target genes are involved are still unknown [13,17,24]. MRTFs, on the other hand, have been shown to mediate the morphogenetic, adhesive, and motile processes [13,24]. It was recently demonstrated that the transcription of much of the serum-induced IEGs is MRTF/SRF-dependent but the role of TCF-SRF signaling could not be determined due to the lack of specific TCF inhibitors as well as the poor quality of TCF chromatin immunoprecipitation (ChIP) results [24,28]. It is generally considered that the TCFs are antagonistic of MRTF-dependent SRF target genes and compete directly for SRF-binding sites. Consequently, this competition is mainly responsible for the balance between the proliferative and contractile gene expressions [24].

## The activation of SRF by interacting with its cofactors

The SRF binding to CArG box enhances its ability to act as an anchoring protein by binding to other cofactors to effect regulation of target gene transcription [29]. However, several mechanisms govern these interactions between SRF and the cofactors, and these remain primarily unexplained [30]. It is a potent transcriptional regulator of target genes, with numerous experiments suggesting over 200 of such genes regulated by SRF [31]. The ability of SRF to regulate these different sets of downstream target genes is a function of the promoter context and its interactions with cofactors [31]. SRF controls the transcription of several IEGs and associates mainly with two families of signal-regulated cofactors, the ERK-regulated TCFs and the Rho-actin controlled MRTFs [30,31]. Some of the mechanisms associated with these SRF interactions with cofactors are briefly discussed here.

### The interaction between SRF and MRTFs

As stated above, SRF transcription is activated mainly by MRTFs when they translocate into the nucleus where they interact with SRF. Therefore, the ability of the MRTFs to regulate this SRF transcription is dependent upon their nuclear translocation [32]. It has been shown that this process is regulated by the RhoA signaling (Rho family of small GTPases) and the subsequent actin polymerization [33,34]. The RhoA signaling increases the F-actin/G-actin ratio in different types of cells through multiple pathways, for instance, by promoting F-actin assembly in fibroblasts and activation of RhoA kinase (ROCK) in vascular smooth muscle cells (VSMCs) [34-36]. This results in the release of myocardin from G-actin and transfers to the nucleus, enabling it to form complexes with SRF to activate the transcription of the downstream genes [37]. This binding is also influenced by RPEL actin-binding domains that enable the MRTFs to bind monomeric G-actin, leading to their retention in the cytoplasm [13,32]. Following stimulation under mechanical stress and actin polymerization into filamentous F-actin, the MRTFs will relocalize to the nucleus with a subsequent increase in SRF transcriptional activity [17,32]. Changes in actin dynamics related to SM physiology also associate with the expression of myocardin [34].

The role of striated muscle activator of Rho signaling (STARS) in promoting nuclear localization of MRTF-A and MRTF-B has also been described, indicating the likelihood of competing with their RPEL motif for actin binding [34,38]. Additionally, the sequestration of MRTF-A results in actin polymerization due to the RhoA signaling pathway and the subsequent activation of SRF caused by the translocation of MRTF-A/MAL from the cytoplasm to the nucleus [17,34,39]. The role of SRF in actin dynamics is responsible for the regulatory loop in which actin synthesis is promoted by changes in cell shape that may influence the cytoskeletal structure [34].

The possibility of a common mechanism regulating SIRT2 and SRF during serum stimulation has also been reported [40]. The *SIRT2* gene is upregulated during conditions of serum deprivation in similar ways that the *SRF* gene also responds to serum deprivation and/or serum restoration following deprivation [40,41]. SRF binding to SIRT2 is associated with a CArG element in the *SIRT2* promoter gene. Here, serum deprivation was reported to induce *SIRT2* expression while SRF and SRF-binding protein, p49/STRAP on the other hand, repressed *SIRT2* expression [40]. The Rho/SRF inhibitor, CCG-1423, also suppressed the expression of the *SIRT2* gene, suggesting that the *SIRT2* gene is a downstream target of the Rho/SRF signaling mechanism [40].

### The interaction between SRF and TCFs

SRF transcription is also controlled by the transcription cofactors TCFs that are activated through MAPK signaling pathways. Recently, a study reported novel results that mitogen-activated cardiac fibroblast utilizes the mechanism related to collagen receptor, discoidin domain receptor 2 (DDR2)-dependent activation of extracellular signal-regulated protein kinase 1/2 (ERK1/2) MAPK, and SRF for coordinated regulation of resistance to apoptosis and cell cycle progression [42]. This is achieved through enhanced expression of apoptotic cellular inhibitor of apoptosis protein 2 (cIAP2) in cardiac fibroblasts with the consequent protection against oxidative injury [42]. Additionally, the transcription process upregulates S-phase kinase-associated protein 2 (Skp2), leading to post-translational degradation of the cyclin-dependent kinase inhibitor, p27, responsible for cell cycle arrest, and promoting G1-S transition, Rb phosphorylation, increased proliferating cell nuclear antigen (PCNA), and flow cytometry [42]. Finally, DDR2-dependent activation of ERK1/2 MAPK also led to the suppression of forkhead box O3, FOXO3a-mediated transcriptional induction of p27 [42].

Interactions between SRF and ETS domain transcription factors have also been reported to be one of the mechanisms for the regulation of the transcription of the *mouse double minute 4 protein* (*MDM 4*) oncogene in hepatocellular carcinoma (HCC) [43]. The MDM 4 protein is known to be a p53-negative transcription regulator that inhibits the transcriptional activities of p53. Its protein and mRNA are upregulated in human HCC due to copy number alterations and post-transcriptional mechanisms associated with the AKT/mTOR signaling [43]. Using in silico analysis, SRF, ELK1, and ELK4 were reported to be putative transcription factors binding to the *MDM 4* promoter region. Also, there was a strong positive correlation between SRF and *MDM 4* expression and high mRNA levels of *MDM4*, *SRF*, and *ELK4* associated with reduced survival of HCC patients following liver resection. On the other hand, inhibition of the transcription factors caused a reduction in the mRNA levels of *MDM 4*, suggesting the critical roles of SRF and its cofactors in promoting the oncogenic function of *MDM 4* in HCC [43]. Therefore, targeting the transcription of *MDM 4* may offer a promising therapeutic approach for the treatment of liver cancer patients [43].

In addition, a new mechanism has been reported involving the repression of the expression of multiple SMC genes by Kruppel-like transcription factor 4 (KLF4) and platelet-derived PDGF-BB [44,45]. First, KLF4 repressed the myocardin-induced activation of SMCs and the expression of myocardin itself [44]. Then, the upregulation of KLF4 under PDGF-BB stimulation reduced SRF binding to CArG-containing regions of intact chromatin [44]. The association suggests that KLF4 represses the expression of SMCs by downregulating expression of myocardin and preventing the SRF/myocardin cofactor interactions in their association with the promoter region of SMCs [44].

### The competition between MRTFs and TCFs and their cell/tissue specificity

The TCFs become phosphorylated when MAPK signaling pathway is activated, and Elk-1 interacts with SRF by binding to the short peptide motif called B-box [34]. On the other hand, myocardin and MRTFs’ SRF binding are similar to the predicted secondary structure of the B-box. However, it differs from that of Elk-1 by the absence of direct amino acid homology [34]. Therefore, the deletion of this myocardin region hinders the ability of myocardin to interact with SRF to activate SRF-dependent target genes. However, these functions are often reversed when Elk-1 B-box replaces this binding region [22,34]. As such, myocardin and Elk-1 compete for this SRF-binding site in a mutually exclusive manner to create a switch that facilitates the regulation of SMCs by growth factors [17,22,34].

Stimulation of SMCs by PDGF results in Elk-1 phosphorylation by MAPK signaling pathway to cause it to interact with SRF and displace myocardin [22,34]. This change in Elk-1 binding to SRF due to repression of myocardin results in an overall reduction in the expression of SMCs because Elk-1 is relatively weaker than myocardin [34]. Conversely, reduction in the levels of endogenous Elk-1 in SMCs will increase the expression of SMC target genes due to derepression of the SRF–myocardin binding [34,46]. Phosphorylation of SRF can also lead to modification and alteration of its affinity for DNA binding [47].

Chromatin immunoprecipitation assay together with human promoter microarrays has been used to identify over 200 SRF-binding sites downstream, including many other new sites in three different human cell lines (Jurkat cells, T/G HA-VSMC, and Be(2)-C cell line) [4]. A genome-wide view of SRF occupancy at its different binding sites with differing cell types was also used along with PCR validations at over half of the binding sites to make deductions of the results [4]. Binding of ELK4 cofactor and epigenetic modifications were reported to be the fundamental mechanisms responsible for tissue-specific SRF binding [4]. ELK4 interacts with SRF to activate the transcription of downstream genes [4]. The interactions of SRF with its cofactors can also be specific to different tissues within the human body [4,48]. It is known that epigenetic mechanisms are critically involved in the regulation of chromatin structure and remodeling, suggesting that they are crucial mediators in cell-type-specific gene expression during growth and disease conditions [49]. Histone modification and DNA methylation are the most extensively studied epigenetic changes. While histone modifications alter the packaging of chromatin, DNA methylation occurs at the 5′ position of the cytosine ring due to DNA methyltransferases (DNMT1, DNMT3A, and DNMT3B) [49].

## The epigenomic regulation of transcriptional control of SRF on their downstream genes

Identifying the genes that SRF regulates is critical to understanding the functional roles it plays in health and diseases [4]. SRF regulation and its target genes demonstrate a typical example of how diverse genes are controlled by a single DNA-binding protein and the significance of cofactors in this molecular regulation of gene expression [34]. Many target genes of SRF regulation are involved in cell proliferation and muscle differentiation, with muscle genes being repressed by growth factor, and are, therefore, not activated until myoblasts are absent from the cell cycle [34].

In a study, several genes were reported to be directly regulated by SRF, with half of them being experimentally validated, and are mainly involved in cell growth, migration, cytoskeletal organization, and myogenesis [34,50]. A common example of SRF target gene that is involved in cell growth is the IEG, *c-fos* which is controlled by SRE, acting together with the surrounding cis elements in the promoter [34]. There is specificity in the expression of CArG box-dependent SRF target muscle genes, with some of the genes being expressed only in one type of muscle cell, for instance, smooth, skeletal, or cardiac muscle cells while others are expressed in multiple muscle cells [34]. Though the molecular mechanisms responsible for this have not been fully elucidated, it has, however, been suggested to possibly involve both positive and negative controls of proteins as well as gene-specific action of SRF [34]. As SRF and MRTFs regulate the transcription of SMC-specific genes through the interactions with the conserved CArG elements within the promoters of the SMCs [20,51], however, the fact that these transcription factors are also present in other non-SMCs demonstrates the possibility of other mechanisms being associated with the expression of these genes [20].

Previous studies have suggested that SRF binding to CArG box DNA sequences within the context of intact chromatin induced the expression of these SMC genes [1,11,19,20]. Chromatin structures determine the permissiveness of DNA sequences to transcription factor binding, and it could offer a glimpse into the regulation of SMCS by SRF [1]. For instance, histone modifications that promote gene expression such as H3 and H4 acetylation, H3K4 methylation, and H3K9 demethylation/acetylation were previously reported at the SMC-specific promoters in SMC [1,10,20]. MRTFs have also been shown to enhance the modification of chromatin by using histone-modifying enzymes [1,20,52]. DNA methylation has been reported to be another mechanism equally responsible for the transcription of SMC-specific genes [20].

The ability of SRF to regulate SMCS also involves other mechanisms that may likely control chromatin structure and access to SMC-specific target gene promoters [1,51]. SRF binding to these target gene promoters has been reported to correlate with positive chromatin marks [51]. Chromatin structure and function are greatly influenced by histone proteins post-translational modifications, and they regulate the permissiveness of chromatin to DNA transcription factor binding by either acetylation or methylation [1]. MRTFs have been shown in several studies to interact with chromatin modifiers [1,51,53,54]. High expression of SRF induced by several agonists, especially transforming growth factor-β (TGF-β), promotes increased SRF binding to the CArG elements present within the promoters of specific genes [51]. In addition to these, phosphorylation of Ser103 by kinases has also been reported to cause increased affinity of SRF to CArG elements [47,51,55].

The association between SRF and CArG is also responsible for the transcriptional repression of these genes during disease conditions owing to changes in environmental conditions both *in vitro* and *in vivo* [1,3]. It is equally essential for cell differentiation and repression under both physiological and pathological conditions, respectively. However, the mechanisms responsible for this association are still not clearly understood [1].

It has been demonstrated in macrophages using genome-wide location analysis that SRF binding is not only enriched at target gene promoters but also occur at distal inter- and intragenic locations [48]. This is contrary to previous studies, suggesting that SRF binding is mainly at the proximal sites because almost all functional CArG boxes were shown to be located within 4 kb of the transcription start site [48,50,56]. Functional studies also established that PU.1, an E26 transformation-specific family of transcription factor, is required to activate these target genes, thereby providing better understanding into the molecular mechanisms regulating cell-specific programs of SRF-dependent gene expression [48].

## Participation of SRF in cellular functions and tissue development

Serum response factor is a highly versatile transcription factor encoded by a single gene that is ubiquitously expressed in different cell types [13]. It regulates the transcription of various target genes that perform diverse essential molecular and biological functions of multiple cells including muscle cells, endothelial cells, fibroblasts, hepatocytes, and neurons. It is involved in the development of gastrulation, heart, vascular system, and liver as well as the immune system and neurons by regulating cell proliferation, differentiation, cell growth, and regeneration [13]. SRF also contributes to the regulation in cell survival [13,57].

By using the strategies of either the downregulation or overexpression of SRF, several cell culture and animal experiments have revealed the significant roles of SRF in serum-dependent cell growth and skeletal muscle differentiation [34,58-60]. In addition, SRF-deficient phenotypes exhibit defective development and maintenance of the heart and GI. However, the survival of the animal varies and may depend on the time of knockout and the promoter that drives the expression of *Cre* recombinase [60]. For example, although SRF knockout has been demonstrated to be lethal in congenital knockout systems, exhibiting cardiac or GI SM defects [5,60,61], *Myh11* knockout of SRF resulted in a more extended survival compared to other promoters such as *SM22α*. Inducible knockout experiments of the genes in adult SMCs also caused severe GI dilation and thinning of the SM layers but survived longer than the congenital knockout model [60]. The phenotypic similarities between congenital and inducible knockout animals suggest the importance of SRF in cardiac and SM development in embryos and maintenance in adults [60].

Furthermore, the importance of SRF in cell growth and skeletal muscle differentiation was also demonstrated in cell culture experiments in which SRF was downregulated [34,58]. This resulted in the blockage of coronary SMC differentiation in chick embryos and disruption of skeletal and cardiac muscle differentiation in transgenic mice [34,59]. Moreover, in a similar way, a homozygous SRF-null mutation in mice had lethal effect at gastrulation, indicating the essential role of SRF in regulating genes involved in cell migration and adhesion needed for gastrulation [8,34].

It is well known that embryonic stem cells that are deficient in SRF exhibit this abnormality due to a loss of actin stress fibers and a consequent loss of the genes associated with components of actin stress fibers such as *vinculin, talin*, and an *actin* isoform [34,57]. Conditional SRF deletion from cardiac muscle led to significant disruption in sarcomeric structure and abnormal muscle gene regulation [5,62,63]. In SMCs, SRF deletion led to reduction in the number of differentiated SMCs near the dorsal aorta, while the few that survived had visible cytoskeletal defects [5,34]. In the skeletal muscle, SRF deletion caused perinatal lethality resulting from hypoplasia [34,64]. SRF may also play a critical role in muscle development; however, the early lethality of SRF-null mice makes the study difficult [34].

Serum response factor is also found to be important for the regulation of the development of axons in the mammalian brain [65]. Conditional knockout mice experiments have demonstrated that SRF plays an important cell-autonomous role in axonal growth [65,66]. Although the mechanisms responsible for these SRF regulatory activities in the neurons are not properly understood, some studies linked it to the phosphorylation of SRF by glucose synthase kinase-3 (GSK-3) which increased SRF binding with MKL1 and MKL2 [65]. More importantly, it was discovered that vinculin (an actin-binding protein and SRF target gene) is involved in promoting axon growth in SRF-deficient and GSK-3-inhibited neurons, suggesting that SRF is important for GSK-3-mediated axonal growth [65]. However, other conflicting reports also showed that blocking GSK-3 activity increases the expression of SRF target genes, suggesting that SRF alone can promote axonal growth in the absence of GSK-3 signaling [65].

In addition, the association of SRF with MRTFs has been reported to be critical for megakaryocyte (Mk) maturation [33]. SRF conditional knockout mice with Mk lineage have been observed to display abnormal Mk maturation and thrombocytopenia, while those with MRTF-A knockout showed blocked Mk maturation [33,67,68]. These conditions become more severe when both MRTF-A and MRTF-B are knocked out in the mice [33,69].

The role of SRF in the regulation of apoptosis has also recently come to light, especially in SMCs where massive apoptosis was observed in a knockout mouse model accompanied by an abnormal increase in apoptotic proteins and a deficiency of anti-apoptotic miRNA [60,70]. SRF depletion/deficiency and inhibition have also been associated with apoptosis in the embryonic heart [62], lung [71], SH-J1 cells [72], and the GI [60]. These studies and others indicate that SRF plays an anti-apoptotic role and is essential for promoting cell survival [60].

## Implications of SRF on various diseases

Since SRF is widely expressed in various cells and regulates numerous genes, it has also been linked with the development of many human diseases [4] (Table 1). In this review, we focused on two of the highest risk diseases: cancer and CVD [4,73-75]. By interacting with its cofactors, SRF controls the expression of most of the genes associated with contractile apparatus and actin cytoskeleton [76,77]. These SRF target genes are involved in numerous processes in the body including contractility, cell movement, and cell growth signaling that are required for the normal development and functioning of the heart and vessels [50,78]. As such, deficiency in the transcription of these SRF-dependent genes can cause various diseases of the cardiovascular system including congenital heart and vascular defects and other cardiomyopathy such as hypertrophy, heart failure, atherosclerosis, and restenosis [13]. Excessive overexpression of SRF may also be pathogenic to the cardiovascular system, suggesting the need for cardiac homeostasis in SRF signaling pathway [13].

Serum response factor inactivation in cells is associated with defective local homeostasis and eventual death in most cases [76]. For instance, its genetic inactivation in developing vascular SMC leads to reduced expression in contractile genes as well as the recruitment of newly developing SMC to the dorsal aorta, eventually causing midgestation arrest of the mouse [5,76]. In SRF knockout mice in the heart-forming region, appearance of rhythmic beating myocytes which is considered to be one of the earliest cardiac defects was blocked, suggesting the role of SRF during early cardiac myocyte commitment and differentiation [5,76].

Mutations in SRF have also been associated with conotruncal heart disease, a group of congenital heart malformations which causes abnormal cardiac outflow tracts [29]. SRF is traditionally known to be a critical factor in heart development, being strongly expressed in the myocardium of the developing mouse and chicken hearts [29]. Loss of SRF arising from inactivation especially during heart development can have lethal consequences and defects in the myocardium of developing mice [29]. The SRF mutants were shown to display impaired SRF transcriptional activity at both the SRF and atrial natriuretic factor promoter, suggesting that they may have potential pathogenic effects [29].

Recent studies have also established a link between upregulation of SRF/myocardin pathways and the pathogenesis of aortic stiffness in age-related hypertension [37,79-81]. Aortic stiffness is known as an independent risk factor for hypertension and cardiovascular morbidity in the elderly, and it is associated with intrinsic mechanical properties of VSMCs [79]. The underlying molecular mechanisms contributing to this condition is not known. Recent studies discovered that the RhoA/ROCK/SRF/myocardin plays a major role in the onset and progression of aortic stiffness and the development of hypertension by mediating a series of alterations including the VSMC intrinsic mechanical property, extracellular matrix (ECM) remodeling, and interaction between VSMC and ECM [37,79-81]. Importantly, these regulations by SRF are specific in VSMCs in large conduct vessel but not in small arteries. Pharmacological inhibition of this signaling pathway selectively attenuates pathological aortic stiffening but did not affect the aortic function in normal condition, suggesting that this could be a novel therapeutic strategy for the treatment of age-related hypertension by targeting these cellular contributors to this condition in the elderly [81].

In Alzheimer’s disease, overexpression of SRF and myocardin in small cerebral arteries was shown to contribute to the pathogenesis of the condition as they increase arterial contractility and reduced blood flow due to the activation of SRF-dependent SM contractile genes [13,82]. It has also been implicated in pathological SMC proliferation in response to injuries leading to atherosclerosis and restenosis [9,13]. Suppression of SRF-dependent gene transcription by the upregulation of other transcription factors such as FOXO4 and KLF4 dedifferentiation of VSMCs also contributes to this phenotypic switch [13,44]. This is because suppression of myocardin and MRTF activities causes SMC proliferation, especially in atherosclerosis and restenosis, suggesting the importance of SRF/myocardin as a sensor under mechanical stress and growth factor signaling to regulate such phenotypic switches in SMCs [13].

Since SRF was found to be involved in the expression of the genes controlling cell proliferation such as *Fos, Junb, Fosb, and Egr1* [4], various studies have associated SRF with tumor formation and cancer metastasis but this role can be either positive, which causes tumor proliferation, or negative, which suppresses tumor cells depending on the specific pathways involved [13,60]. This suggests a dual role of SRF in the pathogenesis of tumor formation [60]. For example, in gastric carcinoma, the promoter and exon 1 of SRF gene become hypermethylated leading to the downregulation of the mRNA expression [60,83]. In colon cancer, abnormal overexpression of a truncated SRF isoform is linked with increased cell survival, suggesting that it may contribute to the pathogenesis of colon cancer though it remains uncertain whether the truncation alone is responsible for induction of cell growth or it simply regulated the effect of SRF [60,84]. The oncogene *four-and-a-half LIM domain 2*, a potent epithelial–mesenchymal transition inducer, has also been implicated in the pathogenesis of cancer cells, especially in prostate and colon cancer. It is a cell cycle and growth modulator that is required for cancer cell invasion, migration, and adhesion to ECM, and its expression is induced by SRF [13,60]. Although there is a relationship between actin/MRTF/SRF circuit with human cancer development, suggesting the involvement of MRTF/SRF neoplastic process, there is no definitive evidence to establish the causative association to clinically reported carcinogenesis [13].

## Conclusion and future directions

As summarized in Fig. 1, the information presented in this review indicates that SRF is a critical transcriptional factor with diverse biological functions in cells and plays an essential role in the development and maintenance of the normal physiological function in multiple important tissues. It is also involved in the pathogenesis of some diseases that cause high mortality. Mechanistically, SRF confers its transcriptional effects by selectively interacting with its distinct cofactors in a cell-specific manner which is regulated by the different upstream signaling of these cofactors. Although there is a lot that still needs to be known regarding the effect of SRF, the evidence from the current study highlights the importance of this factor and brings new insights into the understanding of cellular dynamics of so many functional traits and disease pathogenesis, especially CVD and cancer. These molecular mechanisms of SRF binding and gene transcription regulation can be used as molecular targets for the pharmacological control, intervention, and treatment of these diseases and many other conditions, thus opening new ways and opportunities for future studies.

Based on the known information regarding SRF functions, there are a few critical research areas that need to be addressed. First, since the interaction between SRF and its cofactors is the key determinant of its activity, future research should be focused on the regulatory mechanism that controls this interaction. Although some of the molecular mechanisms regulating the interactions with cofactors TCF and MRTF families have been reported, the controls of these SRF/cofactor interactions are far from fully understood. In addition, other unrevealed cofactors and their functions as well as their biological roles need to be investigated. The efforts on these researches will increase the understanding of the molecular mechanisms underlying the diverse functions of SRF and lead to new strategies to treat the SRF-associated diseases, especially through inhibiting or activating the SRF/cofactor interactions. Secondly, it is notable that SRF plays its role in a cell-specific manner. It is important to discover the mechanisms of the cell-selective effect and their specific regulatory signaling and target genes. These studies will lead to the discovery of the distinct therapeutic targets for different diseases and avoid the side effects due to the broad impacts of SRF and its wide distribution. Thirdly, the binding sites of SRF to its downstream target genes are also not fully identified, and its potential regulatory mechanism remains largely unknown. Particularly, it will be important to investigate the epigenomic network that regulates the binding of SRF/cofactor complex by using advanced techniques to discover the new mechanisms involved in cancer and CVD.

Finally, considering the importance of SRF to the control of numerous biological functions in multiple cells, the development of a novel approach for the prevention of pathological conditions associated with its expression [60] could be of tremendous potential clinical application in the treatment of disease conditions associated with SRF deficiency and overexpression. In addition, some *in vitro* and *in vivo* animal studies have shown that some drug compounds have been found to be effective in the treatment of pathological conditions related to the upregulation of SRF. For example, a group of small-molecule inhibitors of RhoA transcriptional signaling (CCG-100602, CCG-203971, CCG-1423, CCG222740, and CCG222740) has been found to be able to inhibit MRTF/SRF-mediated upregulation of the gene transcription caused by several environmental (mechanical stress) and cytokine (TGF-β) stimuli and repressed fibrosis and ECM stiffness as well as the VSMC stiffness [37,79,81,85-89]. Although the results remain contradictory and the mechanisms involved are still not fully identified, they provide a promising strategy for the development of a therapeutic drug for clinical application. Efforts should be made to explore further the targets of these compounds and the mechanisms involved and the strategies to reduce potential side effects.

## Figures and Tables

**Table 1.** Summary of the studies associated with SRF/cofactors interaction in different diseases.

S/N	Model	Function	Mechanism	Outcome	Reference
1	Cardiac fibroblasts isolated from young adult male Sprague Dawley rats.	Anti-apoptosis and resistance to oxidative injury	ERK1/2 MAPK-activated SRF	Activation of DDR2-mediated ERK1/2 MAPK regulates cell survival and cell cycle progression in cardiac fibroblasts via SRF	[42]
2	Human hepatocellular carcinoma (HCCs)	Liver cancer	Transcription of the *MDM 4* oncogene	SRF, ELK1, and ELK4 were reported to be putative transcription factors binding to the *MDM 4* promoter region and were associated with reduced survival of HCC patients following liver resection.	[43]
3	SRF (−/−) embryonic stem cells	Cell migration	Actin cytoskeletal structure	Downregulation of FA proteins in ES cells lacking SRF led to inefficient activation of the FA signaling kinase FAK and reduced overall actin expression levels in *Srf (−/−)* ES cells. These changes were accompanied by an offset treadmilling equilibrium, resulting in lowered F-actin levels.	[57]
4	SRF knockout mice (cardiomyocytes and SMCs)	Cardiovascular development (growth and muscle differentiation)	Actin contractile and cytoskeletal structure	SRF mutant mice displayed structural defects in the heart and vasculature which coincided with decreases in SRF-dependent gene expression and death.	[5]
5	SRF mutant mice	Skeletal muscle development	Actin cytoskeletal muscle growth and maturation	SRF deletion resulted in formation of muscle fibers without hypertrophic growth after birth leading to death during the perinatal period from severe skeletal muscle hypoplasia.	[64]
6	SRF^−f/f^ mice	Axon growth in mammalian brain	GSK-3-activated SRF phosphorylation	Phosphorylation and activation of SRF by GSK-3 that is critical for SRF-dependent axon growth in mammalian central neurons.	[65]
7	SRF^f/f^ mice	Axon and neuron development	Actin cytoskeleton	SRF mutant mice exhibited deficits in cortical axonal projections with a variable loss of the corpus callosum. The number of proliferative cells in the ventricular zone increased during development. These changes were also observed in the developing excitatory neurons of neocortex and hippocampus.	[66]
8	SMC-restricted *Srf*-inducible knockout mice	Anti-apoptosis	SRF-dependent miRNAs	Mice exhibited severe degeneration of SMCs with reduced expression of apoptosis-associated miRNAs, high level of SMC death, and myopathy in the intestinal muscle layers. These suggest that SMC degeneration via anti-apoptotic miRNA deficiency resulting from SRF deficiency may be responsible.	[61]
9	Cross-sectional study of CTD patients	Heart development	Impaired SRF transcription	Two novel mutations of SRF were identified in the DNA from the peripheral leukocyte cells. There were no differences between the mutants and wild-type SRF in their protein expression and mRNA transcription. However, both SRF mutants had impaired SRF transcriptional activity at the SRF promoter and atrial natriuretic factor (ANF) promoter as well as reduced synergism with GATA4.	[29]
10	SHR and WKY rats	Aortic VSMC stiffening	Extracellular dysregulation (integrin β1 and BMP1/LOX via SRF/myocardin signaling)	Reconstituted vessel segments from SHR VSMCs were stiffer, had different morphologies, and less adaptable to stretch than WKY VSMCs. Also, SHR VSMCs had increased synthesis of collagen and induced collagen in reconstituted vessels in addition to higher levels of active integrin β1 and bone morphogenetic protein 1 (BMP1)-mediated proteolytic cleavage of lysyl oxidase (LOX). These changes were attenuated by an SRF/myocardin.	[79]
11	Alzheimer’s disease patients	Cognitive decline and dementia in Alzheimer’s Disease	SRF/myocardin overexpression	There was overexpression of several SRF/myocardin-regulated contractile proteins with hypercontractile phenotype in AD VSMC. Also, overexpression of myocardin in control human cerebral VSMC caused an AD-like hypercontractile phenotype and reduced endothelial-dependent and endothelial-independent relaxation in the mouse aorta *ex vivo*. However, silencing *SRF* normalized and reversed these changes.	[82]
12	Intestinal cells and human colon cell line	Tumorigenesis	Alternatively spliced variants and isoforms of SRF	Full-length SRF was discovered to be the predominant form of SRF in all 3 cells used (rat IEC-6 cells, normal human colonic mucosa, and HT-29 cells). However, the colon cancer cell lines from poorly differentiated tumors had SRFD5 as the predominant isoform expressed. IEC-6 cells transfected with SRFD5 also had higher survival than the parental cells	[84]

## Abbreviations

ChIP: chromatin immunoprecipitation
cIAP2: cellular inhibitor of apoptosis protein 2
CVD: cardiovascular diseases
DDR2: discoidin domain receptor 2
DNMTs: DNA methyltransferases
ECM: extracellular matrix
Elk-1: ETS like-1 protein
ERK1/2: extracellular signal-regulated protein kinase 1/2
FOXO3: forkhead box O3
GI: gastrointestinal tract
GSK-3: glucose synthase kinase-3
IEG: immediate early gene
KLF4: Kruppel-like transcription factor 4
MAPK: mitogen-activated protein kinase
MDM 4: mouse double minute 4 protein
MKL: megakaryoblastic leukemia
MRTFs: myocardin families of transcription factors
PCNA: proliferating cell nuclear antigen
PDGF: platelet-derived growth factor
ROCK: RhoA kinase
SIRT 1 and 2: sirtuins 1 and 2
Skp2: S-phase kinase-associated protein 2
SMC: smooth muscle cell
SRE: serum response element
SRF: serum response factor
STARs: striated muscle activator of Rho signaling
TCFs: ternary complex factors
TGF-β: transforming growth factor-β
VSMCs: vascular smooth muscle cells
